# Fiber Sorbents – A Versatile Platform for Sorption-Based Gas Separations

**DOI:** 10.1021/accountsmr.4c00201

**Published:** 2024-12-12

**Authors:** João Marreiros, Yuxiang Wang, MinGyu Song, William J. Koros, Matthew J. Realff, Christopher W. Jones, Ryan P. Lively

**Affiliations:** School of Chemical & Biomolecular Engineering, Georgia Institute of Technology, Atlanta, Georgia 30322, United States

## Abstract

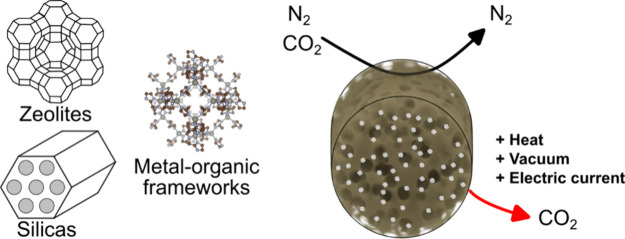

Increasing demand for high-purity fine chemicals and a drive for process intensification of large-scale separations have driven significant work on the development of highly engineered porous materials with promise for sorption-based separations. While sorptive separations in porous materials offer energy-efficient alternatives to longstanding thermal-based methods, the particulate nature of many of these sorbents has sometimes limited their large-scale deployment in high-throughput applications such as gas separations, for which the necessary high feed flow rates and gas velocities accrue prohibitive operational costs.

These processability limitations have been historically addressed through powder shaping methods aimed at the fabrication of structured sorbent contactors based on pellets, beads or monoliths, commonly obtained as extrudates. These structures overcome limitations such as elevated pressure drops commonly recorded across powder adsorption beds but often accrue thermal limitations arising from elevated particle density and aggregation, which ultimately cap their maximum separation performance. Furthermore, the harsh mechanical strain to which powder particles are subjected during contactor fabrication, in the form of extrusion/compression forces, can result in partial pore occlusion and framework degradation, further limiting their performance.

Here, we present the development of porous fiber sorbents as an alternative sorbent contactor design capable of addressing sorbent processability limitations while enabling an array of performance-maximizing heat integration capabilities. This new sorbent form factor leverages pre-existing know-how from hollow fiber spinning to produce fiber-shaped sorbent contactors through the phase inversion of known polymers in a process known as dry-jet/wet quenching. The process of phase inversion allows microporous sorbent particles to be latched onto a macroporous polymer matrix under mild processing conditions, thus making it compatible with soft porous materials prone to amorphization under traditional pelletization conditions.

Sorbent fibers can be created with different geometries through control of the spinning apparatus and process, offering the possibility to produce monolithic and hollow fibers alike, the latter of which can be integrated with thermalization fluid flows. In this Account, we summarize our progress in the field of fiber sorbents from both design and application standpoints. We further guide the reader through the evolution of this field from the early inceptive work on zeolite hollow fibers to recent developments on MOF fibers. We highlight the versatile nature of fiber sorbents, both from the composition, fabrication and structure points of view, and further demonstrate how fiber sorbents offer alternative paths in tackling new and challenging chemical separation challenges like direct air capture (DAC), with a final perspective on the future of the field.

## Introduction

Roughly 50% of the process energy used in the chemical industry is spent on chemical separations.^1^ Growing demand for high-purity chemicals, greater throughput, and improved energy efficiency have driven the search for alternatives or augments to workhorse thermal separation processes.^2^ Adsorption-based separation processes represent a viable alternative, in which chemicals are separated based on different sorbent/sorbate affinities or different molecular diffusivities within a porous adsorbent, and therefore are an energy-efficient alternative, or complement, for existing and emerging separations relevant to the chemical industry.^3^ To date, a multitude of sorbent classes ranging from framework materials (e.g., zeolites; metal–organic frameworks, MOFs; covalent organic frameworks, COFs)^4,5^ to discrete porous molecules (e.g., porous organic cages, POCs; metal–organic cages, MOCs)^6,7^ have been developed bearing a wide variety of chemical functional groups, pore sizes, and pore geometries, offering new and unique possibilities in addressing challenging gas, vapor, and liquid separations. Among the various chemical separations, the world is increasingly focusing on the use of CO_2_ capture to combat anthropogenic climate change. Adsorption-based CO_2_ capture processes are thought to provide some of the lowest overall costs for this globally important technology. This separation pushes the limits on adsorption materials, contactors, and processes.

In a typical adsorption-based separation scenario, chemical mixtures of interest are fed at one end of the adsorption column packed with adsorbent material, forcing the gas mixture components to travel through the column. This approach leads to pressure build-up along the column length due to friction at the boundary between the gas flow and the solid particles, which are typically composed of bound micron-scale powder particles. The resulting pressure drop imposes the use of higher-cost gas management systems (e.g., compressors) and can result in sorbent degradation via attrition and subsequent prohibitive productivity losses over continued operation cycles. This limitation has been historically addressed through sorbent material shaping into macrostructures to maximize sorbent stability and minimize the pressure drop, resulting in energy efficiency gains.^8^ Conventional shaping methods like bead and pellet shaping make use of binders and mechanical force to agglomerate sorbent particulates into macrostructures orders of magnitude larger than the original powders, producing packed beds with pressure drops well predicted by the Ergun equation.^9,10^ Although packed bed systems have been widely deployed for important separations such as xylene isomer purification and air separations, these remain unsuitable for applications that require high throughput and productivity (e.g., direct air capture of CO_2_) due to excessively high pressure drops under high gas flow velocity conditions.^11^

This shortcoming prompted the development of new hybridized sorbent structures in which sorbent particles are deployed as supported pore-bearing fillers to form structured contactors like monoliths and fibers.^12^ These circumvent pressure-drop issues through the arrangement of sorbents in shapes with semi and well-defined flow channels with tunable porosity and pressure drop within the adsorption column, enabling high gas velocities. In addition, such shaping enables a reduction in the characteristic length within the adsorption material, thus simultaneously enhancing mass transport rates relative to pressure-drop-limited packed bed systems. Monoliths are typically prepared by extrusion of a thick, support precursor and binder-containing solution through a die, followed by postshaping processing steps such as solvent exchange and sintering. If the physical and chemical properties of a given adsorbent are compatible with the manufacturing process of monolith contactors, its powder particles can be directly incorporated into the precursor solutions for extrusion, maximizing the volumetric loadings of the adsorbent in the contactor. Alternatively, sorbent particles can be coated onto a monolith’s surface with the compromise of low volumetric loadings. Recently, additive manufacturing (i.e., 3D printing) has been deployed for monolith fabrication with high sorbent loadings and internal microporosity. However, the manufacturing throughput of this technique is limited to a scale of a few centimeters per minute, and thus not yet suitable for mass production of adsorption contactors with dimensions and capacity to address practical applications.

Fiber sorbents present another alternative in the production of shaped sorbents, as these are manufactured by means of robust and mature fiber spinning technology capable of generating fibers at a speed exceeding 50 m per minute for a single filament. Originally established between 2005 and 2010 by Koros, Lively and collaborators,^13−16^ fiber sorbents have exhibited accelerated development, resulting from a wealth of knowledge inherited from the field of mixed matrix membranes (MMM), in which highly robust and selective particle fillers are integrated into polymeric membranes for gas separations. Notable work led by Koros and collaborators on the fabrication and characterization of MMMs led to the identification of critical factors in MMM formation, ultimately paving the way for fiber sorbents.^17−20^ Like monoliths, adsorbents can be coated on the fiber surfaces or loaded into the bulk of the fibers during the spinning step. Engineering the compositions of precursor dope solutions (i.e., a mixture of adsorbent particle and polymer–solvent–nonsolvent system) for fiber spinning allows the optimization of adsorbent loadings while achieving internal interpenetrated macropores via spinodal decomposition for minimal mass transfer resistance. Apart from “solid” fiber sorbents, hollow fiber sorbents can be spun by introducing a bore-forming fluid during spinning to offer additional channels and surface area for process intensification and allowing for direct thermal management when necessary.

To date, fiber sorbents have been the focus of substantial research and industrial interest due to their ease of preparation and scalability, low-pressure drop, high adsorbent loadings, rapid heat and mass transport rates, and potential for process intensification. With this Account we aim to highlight our efforts in the development of fiber sorbents for gas separations with a focus on the following topics: (1) general hollow fiber sorbent fabrication, (2) first-generation zeolite fiber sorbents, (3) amine-infused silica fiber sorbents, (4) MOF fiber sorbents, (5) fiber sorbents for DAC and (6) future perspectives and challenges.

## Hollow Fiber Sorbent Fabrication

**Figure 1:**
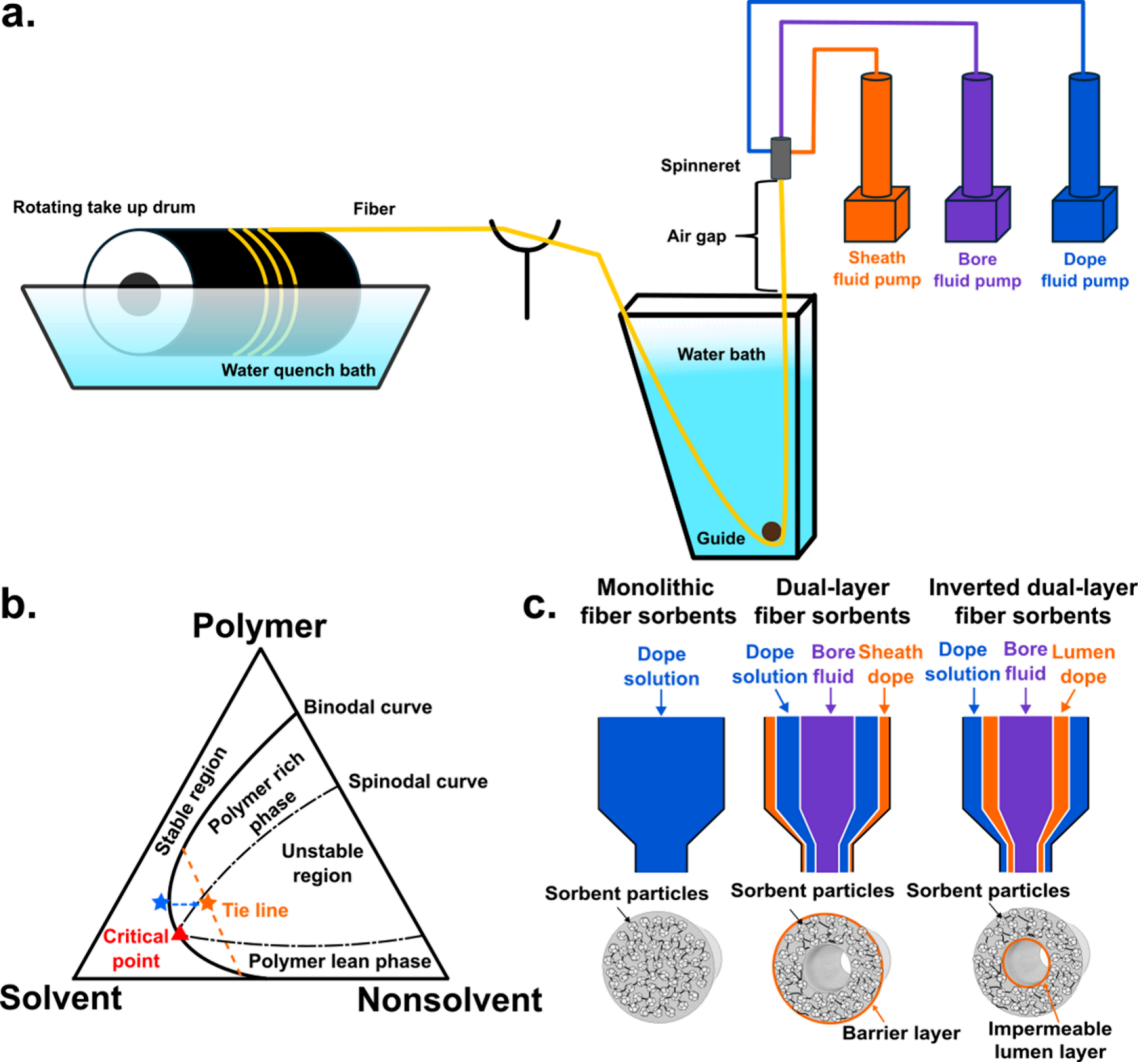
Fundamentals of fiber sorbent fabrication. a. General schematic illustration of dry jet/wet-quenching spinning apparatus. b. Example of a ternary phase diagram representing the ideal scenario of phase transition during fiber spinning. c. Schematic representation of monolithic, dual-layer and inverted dual-layer hollow fiber sorbent structures and corresponding spinneret geometries.

Hollow fiber sorbents are hybrid materials comprised of solid sorbent particles dispersed in macroporous polymetric matrixes with a hollow fiber geometry comparable to mixed matrix membranes. Hollow fiber sorbents are commonly prepared by the well-established dry-jet/wet-quench spinning method historically used for membrane synthesis and originally adapted by Lively et al. for sorbent fiber fabrication.^16^ The process is comprised of several steps (Figure 1a), ranging from dope solution preparation to spinning and postprocessing. Fabrication starts with the formulation of a spinning dope solution (Figure 1b), which is typically extruded through a spinneret (a coannular die, Figure 1c) at high pressures and near-atmospheric temperatures (25–50 °C) by means of syringes or gear pumps. The extruded dope is quenched in a nonsolvent bath (typically water), promoting a nonsolvent-induced polymer phase inversion from liquid to solid, yielding the formation of porous fibers.

Typical polymer dope solutions are made of a spinnable polymer binder, solvent, and nonsolvent. Additional porous materials and additives like salts may also be included for specific applications and fine-tuning of fiber properties. Most polymers used in fiber spinning have published phase diagrams for typical spinning solvents and nonsolvents. Ideal compositions are located near the binodal line of the polymer solution phase diagram, as depicted in Figure 1b, to facilitate rapid polymer phase inversion (ideally spinodal decomposition) upon immersion of the extruded mixture into a nonsolvent quench bath. If necessary, novel dope compositions may be formulated by developing a ternary phase diagram of polymer/solvent/nonsolvent via the use of the cloud point method.^21−23^ As the support scaffold for the fiber structure, the choice of polymer plays a key role in the overall fabrication process and should enable (1) good solution processability, (2) adequate thermal properties, (3) good chemical stability and (4) plasticization resistance. The dope solution is produced by mixing a “prime” dope, whose formulation is based on know-how from the MMM literature, with a dispersion dope. The dispersion dope contains the totality of the sorbent material as well as the remainder of solvent and nonsolvent and can include additives such as pore-forming agents. This approach aims to disperse the sorbent particles in low-viscosity solvents using high-energy methods to break agglomerates; the prime dope is then added to the dispersion dope and physically mixed. This addition increases the viscosity of the resulting dispersion solution, thus inhibiting particle settling.

The final dope solution typically undergoes a period of mechanical mixing, e.g. rolling in a jar, to ensure its homogeneity before it is loaded into a syringe or gear pump for degassing under mild vacuum (∼0.1 bar). The bore fluid, which is needed to produce hollow fibers, is typically prepared by finding a polymer’s “solvent neutrality” point on the polymer solution ternary phase diagram (Figure 1b). Fluids with this composition will not dissolve or phase-invert the fiber from the inside out, which improves spin stability.

Spinning parameters, other than dope composition and quality, can also impact the fabrication process. Variables such as the temperature of the spinning system and nonsolvent bath, the length of the air gap, extrusion flow rate, take-up drum speed, as well as environmental conditions like the humidity and temperature of the surrounding environment can dictate the outcome of the fiber manufacturing process and the quality of the resulting materials.

Once spun, the fibers require adequate postprocessing prior to deployment. Postspinning solution treatments are commonplace when fiber sorbents require impregnation with active compounds, e.g. polyamine impregnated silica fibers.^24−27^ Solvent exchange step(s) may also be undertaken to promote the removal of high surface tension water, the most common nonsolvent, before fiber drying, thus eliminating capillary forces prone to inducing undesirable fiber pore collapse. The exchange solvent generally involves the use of a low surface tension solvent later removed through drying. This step is particularly important in the case of sorbent fillers traditionally requiring thermal activation above the polymer thermal stability range, e.g. zeolites, as it enables gentler activation conditions. If successful, the process yields porous sorbent fibers ready for characterization and application testing.

## Zeolite Fiber Sorbents (First-Generation Fibers)

**Figure 2:**
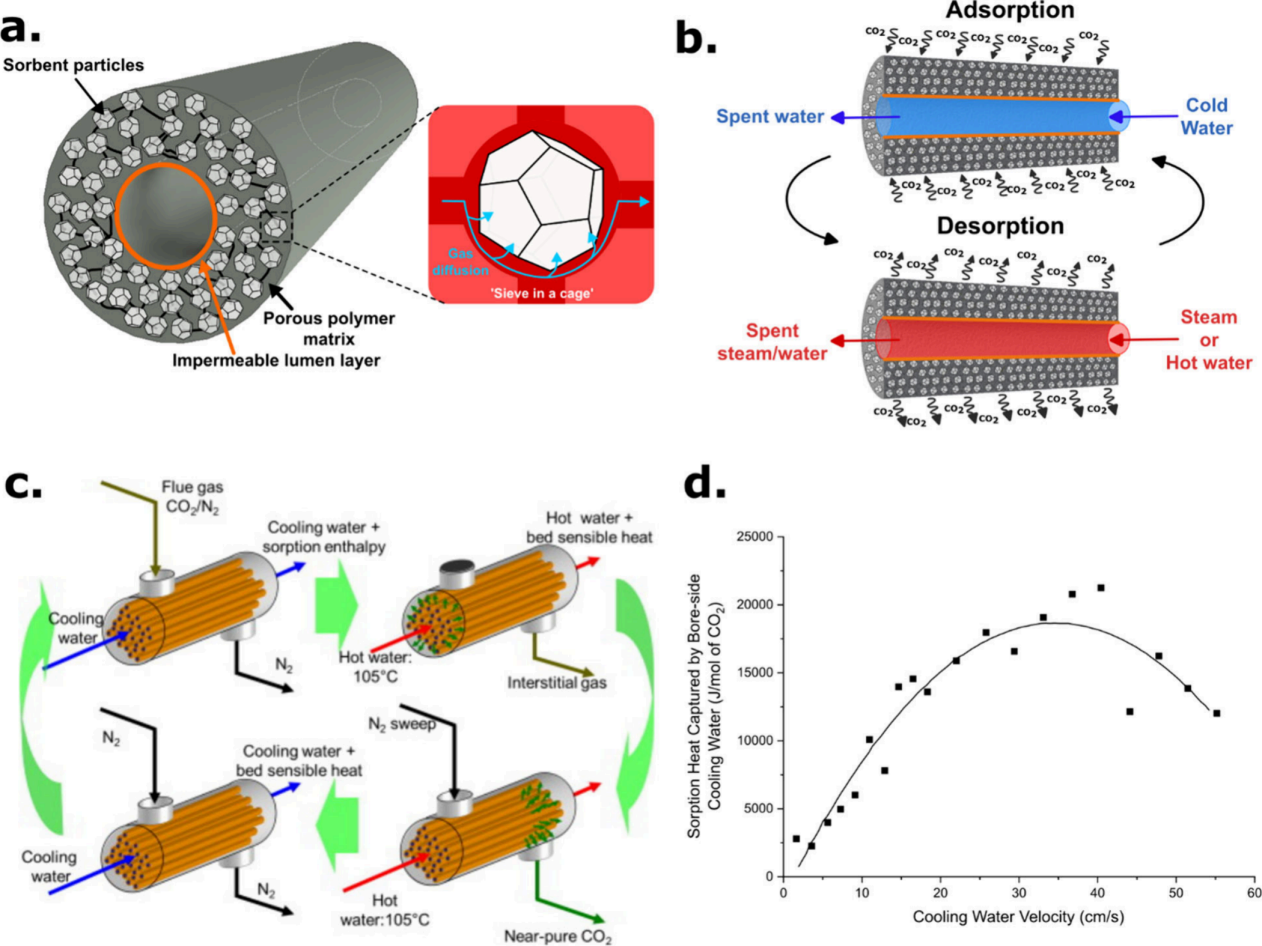
Hollow fiber sorbent structure and sorption principle. a. Structural representation of a porous hollow fiber bearing sorbent particles with inset depicting the ideal sorbent particle/polymer matrix interface (“sieve in a cage”). b. Example of thermal management in hollow fiber sorbent under temperature swing operation. c. Overview of fiber sorbent module in Rapid Temperature Swing Adsorption (RTSA) operation. d. Effect of cooling water velocity on the adsorption enthalpy recovery–fitted experimental results. In this experiment, the adsorption enthalpy released was 36,000 J per mol of CO_2_ captured. Panels c and d reproduced with permission from ref (29). Copyright 2012 Elsevier.

Early fiber sorbent iterations focused on the inclusion of robust microporous zeolites to abate anthropogenic CO_2_ emissions, a field which by the late 2000s saw the common use of energy-demanding, large-footprint liquid sorption or costly single-stage membrane systems (multistage membranes using boiler feed air have ultimately shown to be the low-cost leaders for postcombustion CO_2_ capture from coal facilities^28^). Fiber sorbents offered a paradigm shift compared to these systems. In our original report, zeolite 13X adsorbents with cellulose acetate polymer binder were used to generate hollow fiber sorbents for CO_2_ removal from a model coal-fired postcombustion flue gas. Fiber fabrication efforts resulted in the assembly of a “sieve in a cage” fiber morphology (Figure 2a) holding up 75 wt % 13X particles in place in an open, continuous pore structure allowing for facile gas bulk diffusion throughout the interconnected fiber pore network while avoiding sorbent pore occlusion, in stark contrast with alternative sorbent shaping methodologies reliant on particle agglomeration or densification.^16^ These hollow fibers were further postprocessed to include an impermeable lumen layer. This allowed for direct thermal management by running a secondary fluid (Figure 2b), i.e. cooling water/steam, from the bore side of the fibers during the adsorption/desorption steps of a rapid temperature swing adsorption (RTSA) cycle (Figure 2c).

Examining the thermal performance of hollow fiber sorbents, with and without thermal management, revealed nonisothermal profiles for the latter case. Testing revealed the existence of considerable thermal gradients throughout the fiber sorbents when exposed to high gas velocities (which are ubiquitous in nearly all CO_2_ capture applications), leading to breakthrough capacity losses of up to 40%, at the highest flow rate when compared to the thermalized scenario.^29^ Closer examination found the occurrence of a fast-propagating heat front through the fiber wall, outpacing the CO_2_ gas front, arising from unmanaged sorption enthalpy. These sorption-induced exotherms (i.e., temperature increases arising from gas adsorption) can reduce the efficiency of an adsorption column due to the exothermic nature of the adsorption process. In fiber sorbents, these effects can manifest as thermal gradients, ultimately resulting in capacity losses within the short time scales usually encountered in realistic processes. This can be counteracted by actively cooling the fiber sorbents down, which allows for near-isothermal operation of the adsorption column. The original study estimated that thermal integration could allow the recovery of roughly two-thirds of the thermal energy released via the heat of adsorption (Figure 2d). This heat recovery was found to be a key component of the low energy costs of fiber-sorbent-based processes. Direct thermal management at the fiber level offers expedient thermal equilibration and improved sorption efficiencies but also presents a pathway in addressing parasitic heat load penalties ubiquitous to high throughput bulk gas separations.^30^ While CO_2_ capture remains a prime application for first-generation fiber sorbents, supplementary studies have also demonstrated their potential for hydrogen purification and subambient air separation further highlighting the promise of fiber sorbents in the purification of mixed gas feeds.^31,32^

## Amine-Infused Porous Silica Sorbents (Second-Generation Fibers)

**Figure 3:**
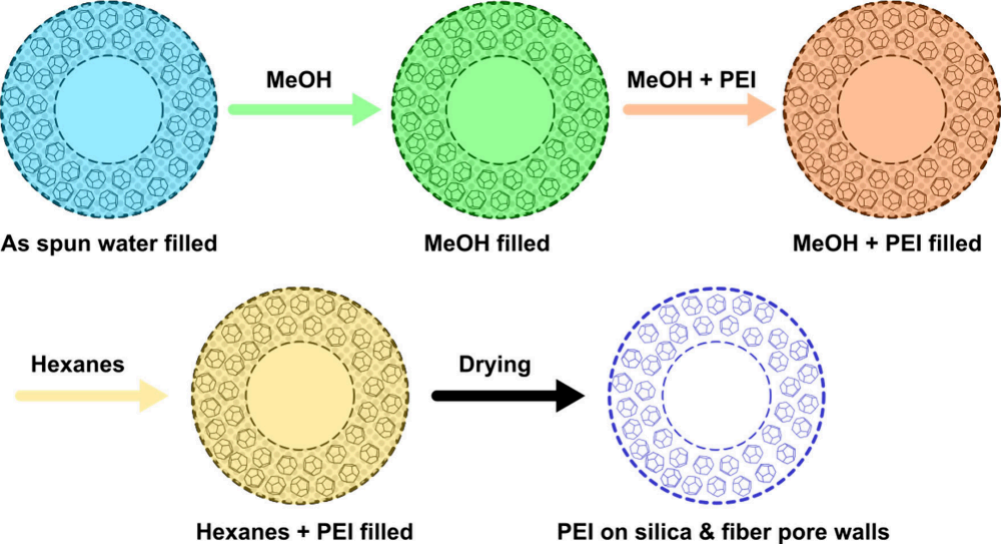
Schematic representation of solvent exchange and amine infusion processing of hollow fiber sorbents. Reproduced with permission from ref (24). Copyright 2013 Elsevier.

With the establishment of zeolite hollow fiber sorbents, attention was shifted to the development of alternate fiber formulations, with the inclusion of commercially available mesoporous silicas as substrates for amine-based CO_2_ adsorbent infusion (class 1 amino silica material).^33^ This approach aimed at exploiting porous silica particles’ structure and chemical environment as support for polyethylenimine (PEI), an amine with CO_2_ capture potential. Hollow fiber sorbents are complementary contactor structures for these materials, as the fiber enables direct thermal management to offset high sorption enthalpies. Amine-impregnated silica-containing hollow fibers were fabricated and tested for CO_2_ capture from a simulated flue gas mixture.^24^ Originally, attempts were made at the direct spinning of fibers containing PEI-loaded silica particles, but these efforts resulted in fiber sorbents with negligible amine content due to leaching during the spinning and postspinning solvent exchange steps. To circumvent this limitation, an alternative postspinning amine infusion strategy was deployed in which fibers containing unfunctionalized mesoporous silica were first spun and then later infused with PEI in a multistep solution treatment process (Figure 3). This postspinning infusion (PSI) approach afforded amine-rich fibers with high CO_2_ uptake capacity of 1.2 mmol/g at 0.10 atm of CO_2_. Beyond this initial study, PSI has been shown to be compatible with numerous fiber formulations,^26,34^ as well as being a viable strategy for the fabrication of class 2 amino silica sorbent fibers.^27^ In the latter case, the PSI strategy was adjusted to fabricate fibers with porous silica particles upon which silylated amine compounds were covalently grafted to the porous silica particles postspinning. The resulting materials were determined to have nitrogen efficiencies over four times higher than analogous fibers obtained through the direct spinning approach. The potential of the PSI approach goes beyond the capacity for active compound infusions allowing also for chemical modification of the sorbent fiber through covalent bonding of active compounds.^27,35^ However, the latter scenario necessitates careful attention due to the complex nature of the PSI functionalization method, as the solvent used in the process must be a nonsolvent for the fiber.

The PSI approach has since become a staple in the preparation of fiber sorbents where the infusion/functionalizing compounds of interest are incompatible with the spinning conditions, with the added benefit of granular control over the composition of the final product—multiple infusion steps may be undertaken to dial in the amine content in the resulting fiber sorbent. Alternative implementations of the PSI method have also shown success in the replacement of deactivated sorbents through fiber reinfusion in instances where the active amine compound was found to undergo degradation or deactivation. PSI is a powerful fabrication strategy that lays a path toward sorbent remediation with the potential to maximize sorbent operation lifetime and process cost minimizations vital to real-world applications beyond the lab scale.

## MOF Fiber Sorbents

The versatility and modularity of sorbent fiber fabrication make it an ideal platform for the deployment of hybrid porous materials such as MOFs, which have thus far seen minimal field deployment due to powder processability and shaping limitations.^36^ The limited intrinsic physical-chemical stability of MOF sorbent fillers can impose fabrication constraints that are absent for zeolite and silica fiber sorbents.^37^ The selection of spinnable MOF materials must first begin with the MOF stability in the presence of water and the mildly alkaline conditions present in the fiber sorbent spin dopes. Fortunately, a variety of MOFs are thermodynamically stable under these conditions, and even some of the kinetically stable MOFs have been found to be sufficiently robust to withstand the fiber sorbent fabrication process without structural loss.^38,39^ An early study by Chen et al. demonstrated the ability to incorporate robust UiO-66 particles into fibers without sacrificing the MOF’s intrinsic sorbent properties.^40^ However, the same cannot be assumed for the vast majority of MOFs that are often prone to the degrading effects of partial or total hydrolysis when exposed to humid conditions, e.g. dry-jet wet-quenching process.^41,42^ This issue is particularly prevalent for MOFs with promising CO_2_ sorption properties such as HKUST-1. To address this issue, a novel fabrication strategy was devised in which macroporous cellulose acetate fibers are spun bearing a robust metal oxide precursor, ZnO, later used as a nucleation seed for the *in situ* generation of the MOFs in question.^43^ Once spun, the oxide-rich fibers can be sequentially exposed to precursor metal salt solutions, either Cu or Zn, followed by the appropriate linkers, benzene tricarboxylic acid (BTC) and 2-methylimidazole (2mIm), resulting in the self-assembly of pristine ZIF-8 and HKUST-1 crystallites. HKUST-1 fibers prepared in this manner were shown to have good CO_2_ capacity, 2.44 mmol/g_fiber_ at high pressures and low-temperature breakthrough conditions (3.5 bar, 0 °C). This “chimie douce” approach, reminiscent of a ship-in-a-bottle assembly, facilitates *in situ* crystallization of quality MOF sorbent particles directly within the confines of the CA fibers’ macropores, circumventing sorbent fiber exposure to conditions known to compromise sorbent quality and performance. In a proof-of-concept study, Pimentel et al. further demonstrated the potential of CA/ZIF-8 monolithic fibers, containing large micron-sized MOF particles, for kinetic separation of propane/propylene in a vacuum swing adsorption cycle (VPSA), highlighting improved energy efficiency over reference cryogenic separation methods by as much as 42%.^39^ Similarly, CA/MIL-101(Cr) fibers have been shown as viable sorbent media for PVSA applications such as CO_2_ capture from flue gas under subambient conditions. Under these operating conditions, monolithic MOF fibers showed very high breakthrough and pseudo equilibrium capacities, 4.4 and 8.8 mmol/g_fiber_, respectively, yielding high-purity desorbed CO_2_ gas feeds.^44^

The earlier success of the PSI method prompted the development of amine-impregnated MOF fibers for CO_2_ capture from dilute streams.^45^ We explored the potential of spinning Mg_2_(dobpdc) (dobpdc^4–^ = 4,4′-dioxidobiphenyl-3,3′-dicarboxylate) in poly(ether sulfone) (PES) hollow fibers. In this work, a direct spinning route was attempted to streamline the first demonstration of fiber sorbents based on this MOF which required a strenuous multistep procedure.^46^ This first account of direct spinning of an open metal site MOF, Mg_2_(dobpdc), offered a viable platform for amine immobilization onto the unsaturated metal sites of the porous MOF. In this process, we successfully fabricated hollow fibers rich in MOF sorbent, which were later infused with 2-(aminomethyl)piperidine (2-ampd). The infused fibers showed isotherm and breakthrough profiles consistent with pure 2-ampd-Mg_2_(dobpdc), in powder form.

## Fiber Sorbents for CO_2_ Capture from Ultradilute Streams

The bench-scale success of hollow fiber sorbents for postcombustion CO_2_ capture prompted the examination of fiber sorbent performance under even more dilute adsorbate conditions, namely CO_2_ removal from air or direct air capture (DAC). The lower CO_2_ concentration and adsorption rates observed under ultra dilute conditions makes viable the use of monolithic fibers that are devoid of an open bore for thermal integration (i.e., the adsorption-induced exotherm is largely negligible in DAC). With this in mind, porous monolithic CA/silica/PEI fibers and testing methodologies for DAC applications were explored.^47^ These fibers, bearing mesoporous silica particles, were fabricated and postspinning infused with PEI for operation in a temperature swing adsorption (TSA) cycle. The fibers were deployed in a module similar in design to those used previously for postcombustion systems, but devoid of bore fluids feeds in this case. Thermal management was instead performed through direct heating of the module enclosure to promote gas desorption during cyclic testing without need for conventional desorption gas sweeps commonly found in the literature. The study went on to examine the impact of key variables like feed humidity and superficial air velocity in CO_2_ capture performance with CA/silica/PEI fibers. Preliminary examination revealed that the densely packed fiber module outperforms small particle PEI/silica packed beds regarding pressure drop, as expected. The direct comparison to packed beds, well described by the Ergun equation, revealed significantly lower pressure drops at higher superficial air velocities (>1 m/s), for which the deployment of packed beds is nonviable. The sorbent fibers were found to be highly sensitive to testing temperature when testing for the adsorption step in the range of 35–55 °C, with considerable drops in recorded breakthrough and pseudo equilibrium capacities at elevated temperatures, as is to be expected whenever CO_2_ adsorption is thermodynamically governed. Further evidence of good sorbent accessibility and fast gas diffusion kinetics into the fiber phase was unveiled through testing with higher gas velocities (40–90 cm/s), which showed equally sharp breakthroughs while maintaining the same pseudo equilibrium capacity throughout. A follow-up study by Kong et al. recorded a near tripling in CO_2_ capacity for CA/silica/PEI fibers when exposed to an 85% RH humid feed (lab air), at 20 °C, compared to dry feed conditions (simulated air).^48^ Fiber performance was found stable under these conditions after an initial cycle, as is common for this class of sorbent fibers. Cyclic testing revealed water adsorption can reach levels nearly 1 order of magnitude higher than CO_2_ (6 mmol_H_2_O_/g_fiber_ vs 0.7 mmol_CO_2__/g_fiber_) further underscoring the increased thermal duty when operating these sorbents under humid conditions. The study went on to evaluate the impact of parameters like PEI loading and superficial airspeed on the overall sorbent productivity, a critical metric for efficient field deployment. A delicate balance between PEI loading, CO_2_ capacity and sorption kinetics was observed–fibers with more PEI offered more CO_2_ adsorption but also faster breakthroughs and slower pseudo equilibrium times, suggestive of greater internal mass transport limitations which can negatively impact sorbent productivity—superior kinetics often outweigh the importance of a moderately higher CO_2_ capacity. Ultimately, a maximum productivity of 1.42 mmol_CO_2__/g_fiber_ per hour was determined possible, assuming a desorption and cooling phases of 5 min made possible by the sorbent fibers’ optimized heat and mass transfer performances.

**Figure 4:**
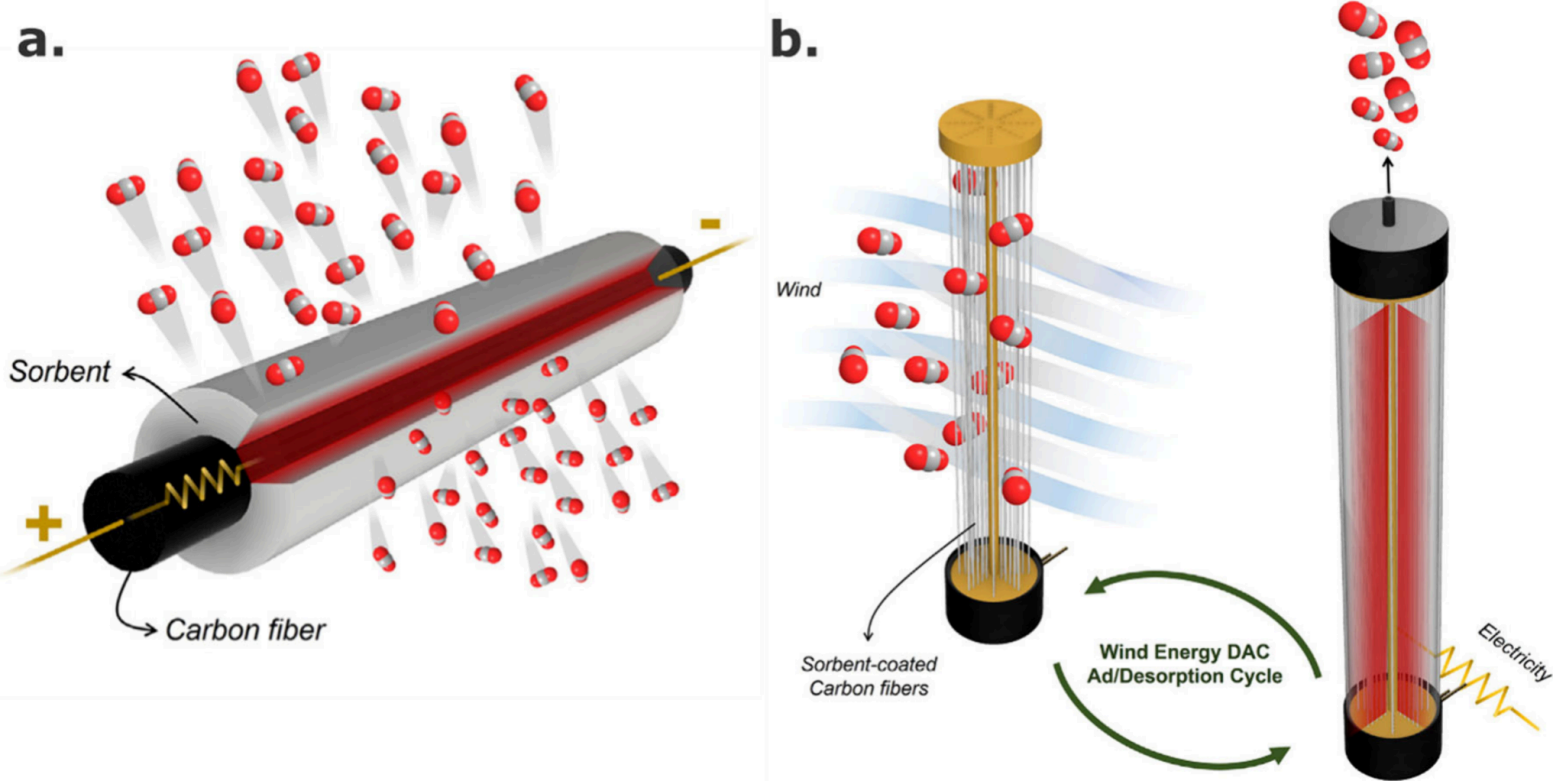
Representation of a CA/silica/PEI sorbent fiber coating on an electrically resistive carbon fiber for ETSA. Reproduced with permission from ref (49). Copyright 2023 Elsevier.

Resistance heating methods have recently been proposed to improve CA/silica/PEI fiber sorbent productivity.^49^ Porous sorbent fiber coatings were integrated with an electrically resistive carbon fiber core resulting in a resistive heating CO_2_ sorbent system with improved heating/cooling kinetics and high thermal efficiency. The integration of a sorbent shell with a solid thermal contactor core enabled fast, uniform thermal cycling of the DAC module, i.e., 25–120–25 °C in 100 s, in a first account of electric thermal swing adsorption (ETSA) with fiber sorbents (Figure 4). When compared to more common external heating TSA approaches, this internal heating method not only led to faster thermalization kinetics as result of localized thermal energy generation but also offered more effective heating energy usage as less thermal energy was dispensed on sensible heating of the DAC module, thus allowing for faster process times that are beneficial to unit productivity. A techno-economic analysis of this unique system revealed most of the process cost was attributed to the electric consumption during the desorption step. Nonetheless, an estimated levelized cost of CO_2_ of $160 tCO_2_^–1^ was determined in stark contrast to costs reported for currently deployed DAC technologies ($300–$800 tCO_2_^–1^),^50^ made only possible through the deployment of fiber sorbents. If coadsorption and desorption of water is efficiently managed, then an estimated levelized cost of CO_2_ was estimated to be $160 tCO_2_^–1^ for this coated fiber sorbent system, which is significantly lower than reported costs for currently deployed DAC technologies.

## Future Outlook

The ever-growing need for energy-efficient separation processes has driven the development of structured sorbent materials in recent years with new and exciting prospects for process intensification. Fiber sorbents have been identified as one enabling option, particularly in the field of gas separations, as briefly presented in our summary overview of the field. In our opinion, some key challenges remain unaddressed, which we will now outline to guide future fiber sorbent research.

**Figure 5:**
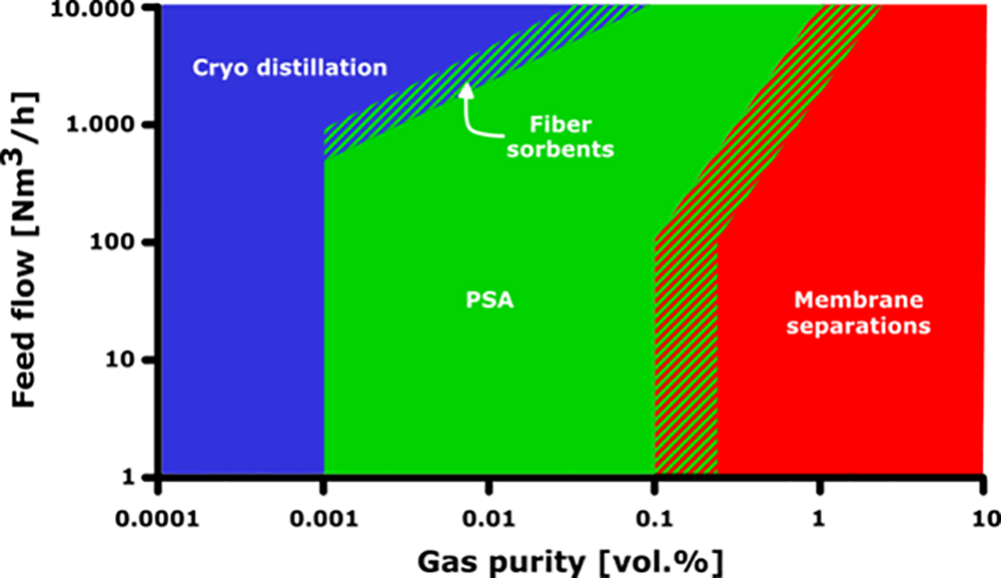
Conceptual selection diagram for product gas purity, including fiber sorbents.

Despite growing interest, most fiber sorbent reports remain limited to the lab scale. While the polymeric membrane fiber industry is well established, as noted above, the development of large-scale mixed-matrix membranes or composite polymer/filler sorbents, as described here at the lab scale, has not been explored for large-scale deployment based on publicly available information. However, the intense interest in CO_2_ capture, and specifically DAC, has driven high throughput adsorptive separation process research and development, an application area in which fiber sorbents can excel. Figure 5 gives an example of how the increased productivity and throughput of fiber sorbent systems relative to traditional pellet-packed bed systems broadens the potential operating window of adsorption as a separations technology.

Beyond the commercial availability of these composite fiber materials, the establishment of detailed techno-economic and scale-up studies, considering not only performance but also ancillary parameters like maintenance, sorbent lifetime, etc., remains a necessity in identifying key scaling limitations and process optimization requirements. We also note the need for standardized testing and reporting guidelines in shaped sorbent studies, enabling fair and grounded comparisons of different structured sorbent technologies. This issue often adds to the difficulty of assessing the performance of different shaped sorbents and results in inefficient, duplicate examinations seeking head-to-head comparison of different sorbent systems. The issue is further aggravated in the case of fiber sorbents, as a multitude of testing conditions can be found in the literature, i.e. different combinations of polymers and sorbents tested for different separation applications under distinct conditions. Therefore, we propose the establishment of a) a standardized library of results for ease of analysis and b) reference testing conditions minimizing experimental variations and reporting metrics across the resulting studies.

Continued expansion of the portfolio of sorbent materials available for fiber fabrication is another area where considerable progress is to be expected. As new classes of highly tailored sorbent materials achieve maturity, allowing for their large-scale, low-cost production, promising new formulations are expected to find their way into the field of fiber sorbents. Tunable, soft, porous materials like porous organic cages (POCs), covalent organic frameworks (COFs) and metal–organic polyhedra (MOPs) have shown great promise for gas separation applications and may represent future areas of advancement in fiber sorbents.

**Figure 6:**
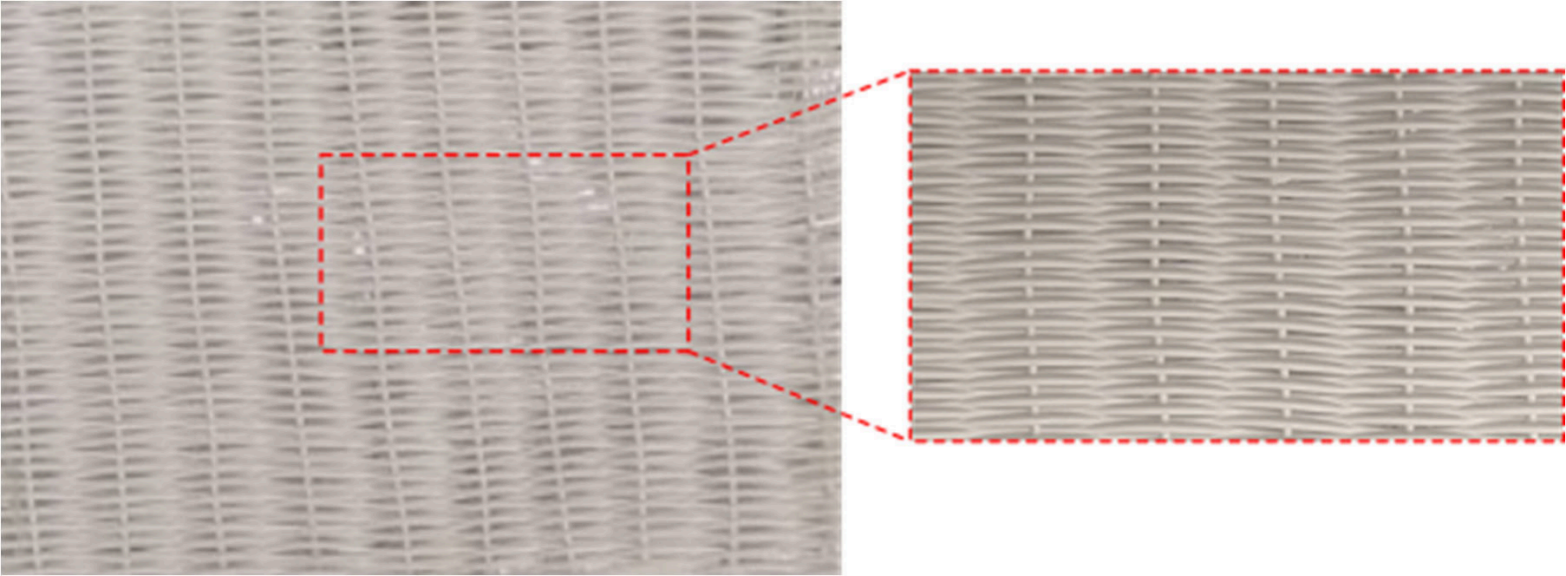
Example of a sorbent fiber woven laminate prepared in the Lively lab.

Contactor design is another area worthy of focus for future development. Most reports rely on the use of fiber sorbents in small radial tube contactors reminiscent of membrane modules. However, fiber sorbents offer the possibility of woven and nonwoven fabric/mat fabrication which has thus far remained unexplored in literature, to the best of our knowledge (e.g., Figure 6 shows a woven fiber sorbent mat prepared in our lab). These higher-order structural arrangements can enable the deployment of fiber sorbents in otherwise difficult-to-implement applications like gas filters or laminate contactor geometries. Other potential benefits may include easier fiber handling for large-scale processability and straightforward integration of fiber sorbents with traditional fin and plate heat exchangers whenever advanced heat integration may be required.

A general area of interest is the long-term stability of fiber sorbents, with limited data available due to the lab-scale nature of most literature studies. Nonetheless, aging studies are necessary to understand long-term use and to enable lifetime assessments, particularly so when considering the polymeric fiber content of the materials as glassy polymers are prone to plasticization and aging. It remains unclear how these issues may impact the fiber performance as well as how sorbent particles age within the confines of the polymeric support matrix, or if/how these effects can be mitigated, an issue particularly relevant in case of MOF fibers given that most MOF structures are thermodynamically unstable and can naturally undergo defect formation and/or structural modification over time (e.g. defect cluster formation in ZIF-8 under acidic gas exposure,^51^ or thermal degradation of HKUST-1 when exposed to temperatures of 300 °C and higher).^52^ Fiber aging can also result in morphological changes and in turn modified mechanical properties, bearing the potential risk of fiber rupture or failure, which can lead to performance losses and reduced separation efficiencies. While several studies can be found on the examination of the mechanical properties of polymeric fibers, with established strategies for mechanical improvement, no such reports were found for sorbent fibers. The need for such systematic studies is further heightened when considering fiber spinning processes and operating conditions–most fiber activation procedures in any process cycle require an energy input (thermal or vacuum) which incurs in mechanical strain, in turn potentially leading to material fatigue and breakage. Therefore, there is an impending need to bridge the gap between nano- and macro-scale physical properties for the design of better sorbents.

Finally, it is worth considering what the next frontier will be for fiber sorbents. A wide array of separation challenges exist, and in general, fiber sorbent technologies will be most advantaged in high-throughput gas separation applications. However, much of the literature—if not the entire body of literature on this topic—has focused on relatively low-temperature separations. Developing fiber sorbent structures for operation at elevated (>100 °C) and extreme (>300 °C) temperatures is needed to continue to broaden the utility of these functional materials. As a final note, it is worth highlighting that this report is limited to dry-jet/wet-quench fabrication of fiber sorbents and that other noteworthy fabrication methods, like electrospinning,^53−55^ are expected to offer unique opportunities for fiber sorbent development in further development of this field.
